# Ki-67 and CD100 immunohistochemical expression is associated with local recurrence and poor prognosis in soft tissue sarcomas, respectively

**DOI:** 10.3892/ol.2013.1226

**Published:** 2013-03-05

**Authors:** MARCELO CAMPOS, SILVANA GISELE PEGORIN DE CAMPOS, GUILHERME GOMES RIBEIRO, FLÁVIA COLTRI EGUCHI, SANDRA REGINA MORINI DA SILVA, CLEYTON ZANARDO DE OLIVEIRA, ALLINI MAFRA DA COSTA, EMÍLIO CARLOS CURCELLI, MARCOS CEITA NUNES, VALTER PENNA, ADHEMAR LONGATTO-FILHO

**Affiliations:** 1 Molecular Oncology Research Center, Barretos Cancer Hospital, Pio XII Foundation, Barretos 14780-000;; 2 Faculty of Medicine, São Paulo State University, UNESP, Botucatu 18618-970;; 3 Laboratory of Medical Investigation 14, Faculty of Medicine, University of São Paulo, São Paulo 01246-903, Brazil;; 4 Life and Health Sciences Research Institute, School of Health Sciences (ICVS), University of Minho, Braga 4710-057, Guimarães, Portugal

**Keywords:** soft tissue sarcomas, Ki-67, CD100, recurrence, poor prognosis

## Abstract

Soft tissue sarcomas (STSs) are a heterogeneous group of mesenchymal tumors of >50 subtypes. However, STSs represent <1% of types of cancer. Despite this low frequency, the disease is aggressive and treatment, when possible, is based on traditional chemotherapies. A number of cases of resistance to adjuvant therapies have been reported. Metastases are commonly identified in STS patients during diagnosis and the development of effective clinical parameters is crucial for correct management of the disease. The use of biological markers in cancer is a useful tool to determine patient prognosis. Ki-67 is a protein marker for proliferation of somatic cells and is widely used in prognostic studies of various types of tumor, including STSs. Cluster of differentiation 100 (CD100) is a member of the semaphorin family. The family was initially described as axon guidance molecules important for angiogenesis, organogenesis, apoptosis and neoplasia. CD100 was previously utilized as a prognostic factor in tumors and also in STSs. In the present study, protein expression of Ki-67 and CD100 was analyzed by immunohistochemistry in samples of STS patients of the Barretos Cancer Hospital (Barretos, Brazil) to establish prognostic criteria of the disease. Results demonstrate a correlation between CD100 expression and poor prognosis, consistent with a previous study. Moreover, the expression of Ki-67 was identified to correlate with presence of local or locoregional recurrence. To the best of our knowledge, no large casuistic study has revealed this correlation between Ki-67 and local recurrence in STSs. The use of Ki-67 and CD100 as markers in clinical pathological analysis may be suitable as a prognostic criterion in disease progression.

## Introduction

Soft tissue sarcomas (STSs) are a heterogeneous group of malignant neoplasias of >50 subtypes of mesenchymal origin. These tumors represent <1% of all types of cancer and their classification is based on histological morphology of the tumors (1). STS incidence is low, annually affecting ∼200,000 individuals worldwide. However, these neoplasias are aggressive and the treatment, when possible, is based on traditional chemotherapies often associated with resistance. STS cells have been identified to originate from various cell lines, which explains, in part, the variety of phenotype characteristics observed in this tumor (1–3).

Metastases in STS patients are frequently identified at diagnosis and consequently, the correct utilization of treament parameters during disease managment is important (4). Histological grade, size, depth of tumor and status of surgical resection are currently used as prognostic factors for STS. Nevertheless, histological grade is currently the best criterion to determine tumor aggressiveness (2). At present, various molecules associated with the biological behavior of these tumors have been described for STSs and may improve clinical diagnosis (4).

Ki-67 is a well-known protein located in the nucleus and nucleolus of cells and is associated with proliferation of somatic cells. Ki-67 is absent in quiescent cells (5). For this reason, human Ki-67 protein is an efficient immunohistochemical marker for establishing levels of malignant cell proliferation and is widely used in the diagnosis of several types of human tumor, including STSs (6). High expression of Ki-67 protein in STSs, is frequently associated with poor prognosis (7), including the occurrence of distant metastases (8).

Cluster of differentiation 100 (CD100) protein is an additional immunohistochemical marker of various types of tumor. CD100, also known as Semaphorin 4D (Sema4D), is a homodimeric glycoprotein markedly expressed in lymphoid tissue, skeletal muscle and at lower levels in the human brain (9). CD100 has been identified in two forms, membrane-anchored and soluble. Membrane and soluble forms function as a receptor with high-affinity to Plexin B1 or a ligand to the low affinity receptor, CD72, respectively (10,11). Semaphorins were primarily classified as axon guidance molecules (12–14). However, this family is also involved in angiogenesis, organogenesis, apoptosis and neoplasia (15–18), as well as in human immune responses (19) where CD100 functions as a ligand or receptor to modulate the activities of B and T lymphocytes. In addition, CD100 interaction with Plexin B1 induces migration and tubulogenesis of endothelial cells (20). CD100 is involved in a molecular pathway with Plexin B1 and Met to promote invasive growth of malignant epithelial cells (21). Ch’ng *et al*(22) previously reported a correlation between CD100 and poor prognosis in STSs. Accordingly, CD100 is involved in various mechanisms of tumor progression, including angiogenesis, invasive growth and regulation of tumor-associated macrophages (23).

The aim of the present study was to evaluate Ki-67 and CD100 expression in STS patient samples from Barretos Cancer Hospital (Barretos, Brazil) to determine whether, in the current population sample, reproducible results of the markers as effective prognostic factors in STSs are obtained. Results demonstrate that CD100 is an indicator of poor prognosis in STSs, consistent with the results of Ch’ng *et al*(22). Furthermore, Ki-67 expression in these tumors was identified as an effective prognostic tool capable of predicting local recurrence. To the best of our knowledge the current study is the first description of the use of Ki-67 as an indicator of local recurrence in STSs.

## Materials and methods

### Patients

Sixty-five patients with STSs were treated at the Department of Orthopaedic Oncology of Barretos Cancer Hospital (Barretos, Brazil) between 2000 and 2009. Of these patients, 42 (64.6%) were male and 23 (35.4%) were female. Patients were predominantly Caucasian (43, 66.2%) and 38 (58.5%) were from São Paulo. Among the patients, 36 cases (55.4%) came to the Hospital following previous treatment and 35 cases (53.8%) arrived with advanced disease (M1). Of the individuals who arrived with M1, the lung was the most frequent site of distant metastasis, representing 31 cases (88.6%). The average lag time was 15 months (SD=19.7). Only one tumor was classified as benign neoplasia. The tumors were located in the lower and upper limbs at a frequency of 44 (67.7%) and 21 (32.3%) patients, respectively.

Tumors were classified as low grade, histological grades I and II (41.5%); and high grade, histological grade III (58.5%). Chemotherapy was performed in 32 patients (49.2%) and radiotherapy in 33 patients (50.8%). Resection was the most common surgery and was performed in 44 patients (68.8%), followed by amputation in 20 patients (31.3%). One patient did not undergo a surgical proceedure. During the follow-up, 28 patients died (43.1%) and 32 relapsed (49.2%). Of all recurrences, 24 (75.0%) had local recurrence. Mean patients follow-up of was 45 months.

### Histological samples

Paraffin blocks of tumor samples were obtained from 65 patients diagnosed with STS from the Department of Pathology and treated by Orthopaedic Oncology (Barretos Cancer Hospital). Areas free of tumor involvement were used as control samples. The confirmation of the quality and the pathological diagnosis of all samples was performed by a pathologist. Following selection of cases, socio-demographic and clinical data were collected from patient medical records to characterize the samples. The distribution of the histological classification of tumors was performed according to the World Health Organization (1) and is depicted in Table I.

### Immunohistochemistry

Following deparaffinization and rehydration of samples, antigen retrieval was performed using citrate buffer (10 mM, pH 6.0) for 30 min in a Pascal pressurized heating chamber (Dako, Carpinteria, CA, USA). Following cooling, the tissue samples were blocked in endogenous peroxidases (3% hydrogen peroxide in methanol). Subsequently, the material was incubated with normal horse serum or serum-free protein (Dako) for 1 h to block non-specific protein. Primary antibodies against the protein Ki-67 (Dako) and CD100 (Abcam, Cambridge, MA, USA) were applied to the samples according to the manufacturer’s instructions, using the following dilutions: Ki-67 (1:100) and CD100 (1:100), with overnight incubation at 4°C. Following this, peroxidase-conjugated secondary antibody (Abcam) or the amplifier polymer ADVANCE HRP detection system (Dako) were applied to the samples according to the manufacturer’s instructions. Antibody binding was visualized using chromogen diaminobenzidine (Sigma-Aldrich, St. Louis, MO, USA) and counter-stained with hematoxylin. Positive controls for each primary antibody were used according to the manufacturer’s instructions.

### Evaluation of CD100 and Ki-67

CD100 was evaluated on a scale from negative, +, ++, +++ and the stained surface area was calculated (stained surface area varied between 20 and 100%). For Ki-67, only nuclear reaction was considered positive. Positive immunostaning was graded as follows: negative (0); faintly positive, between 1 and 10 (+); sporadic, >10 and 50 (++); and diffuse, >50 cells (+++) were positive. In statistical calculations, positive reactions of the markers were considered as positive or negative.

Images of histological sections were captured using the Eclipse 50i microscope coupled to a Sight DS-FI1 digital video camera (both from Nikon, Tokyo, Japan) and analyzed using the Image-Pro Express (v6.0; Media Cybernetics, Rockville, MD, USA).

### Statistical analysis

Variables considered in this study for statistical analysis were: ethnicity, gender, histological grade, survival at 3 and 5 years and expression of CD100 and Ki-67. To characterize the study sample, data were summarized in terms of mean, standard deviation, median, minimum and maximum, when the variables were quantitative; and frequency and percentage for qualitative variables. Global, disease-free and local or locoregional disease-free survival were compared with the events, death from any cause, local recurrence and local and/or regional recurrence, respectively. Initially, the survival rates were estimated using a nonparametric Kaplan-Meier estimator. The log-rank test was used to verify the difference between survival curves for various strata of the same variable. Following this, we used the Cox’s multiple regression model to determine the effect of combinations of variables. Data were correlated and analyzed with SPSS software (for Windows, v19.0). P<0.05 was considered to indicate a statistically significant difference.

### Ethics

The present study was submitted to the Ethics Committee in Research (Barretos Cancer Hospital; no. 331/2010). Informed consent was not required as the study was a retrospective cohort.

## Results

### Expression of Ki-67 and CD100 markers in STS

**Figure 1:**
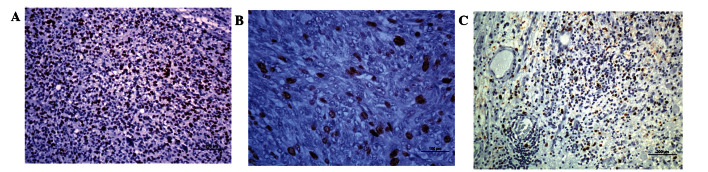
Immunohistochemistry for Ki-67. (A) High grade pleomorphic undifferentiated sarcoma, (B) high grade undifferentiated sarcoma (C) metastatic sinovial sarcoma.

**Figure 2:**
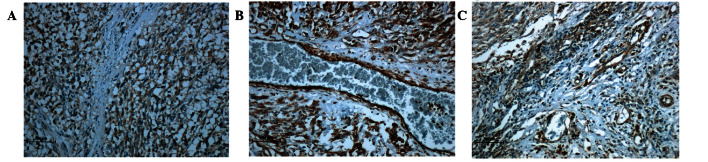
Immunohistochemistry for CD100. (A) High grade indiferenciated pleomorphic sarcoma, (B) leiomyosarcoma grade III, (C) sclerosant epithelioid fibrosarcoma. CD100, cluster of differentiation.

Labeling of Ki-67 was negative in ∼40% of tumor samples. Marker expression was considered low, moderate and severe in 27.7, 18.5 and 13.8% of patient samples, respectively (Table II, Fig. 1). Patient samples (∼18.5%) were negative for CD100, 24.6% had moderate and weak expression (each group) and 32.3% of the samples were identified to exhibit marked expression of the molecule (Table II, Fig. 2).

### Univariate and multivariate analysis for prognostic factors in STS

**Figure 3:**
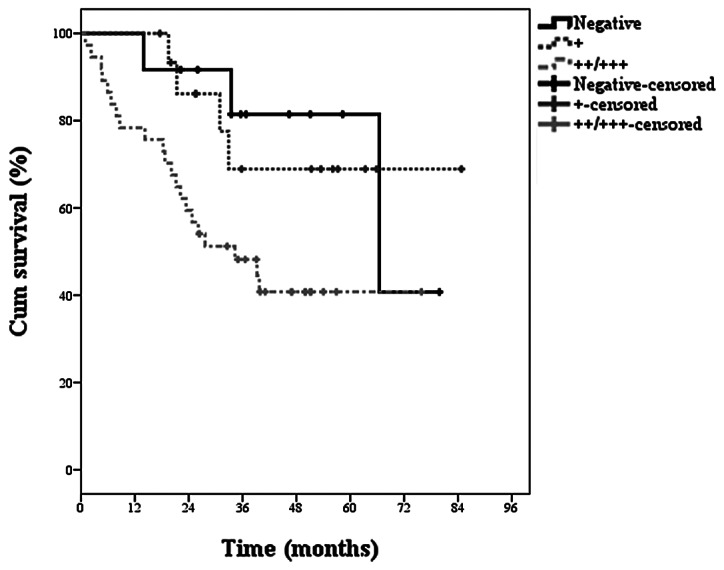
Kaplan-Meier estimate for global survival with respect to expression of CD100. CD100, cluster of differentiation.

The histological grade of tumors was statistically significant (P= 0.001) for the risk of death (global survival) in patients. Similarly, our results demonstrated a statistically significant correlation (P=0.037; Table III) between the increased expression of CD100 and decreased survival (Fig. 3). By contrast, the analysis of Ki-67 expression in tissues was not statistically significant when considering the overall disease-free survival. When evaluating additional variables, including chemotherapy, radiotherapy, surgery type, gender, ethnicity and previous treatment, any statistical significance was identified by an increase in overall survival.

In the assessment of local disease-free survival (Table IV) there was statistical significance for histological grade and expression of CD100. Again, the analysis of expression of Ki-67, was unable to demonstrate the significance of local disease-free survival. In addition, other variables, including chemotherapy, radiotherapy, surgery type, gender, ethnicity and previous treatment do not show statistical significance.

**Figure 4:**
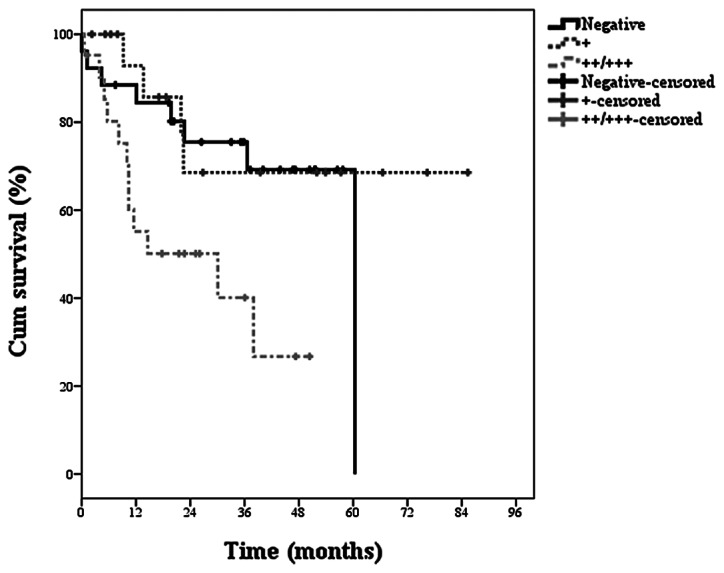
Kaplan-Meier estimate for global survival with respect to expression of CD100. CD100, cluster of differentiation.

**Figure 5:**
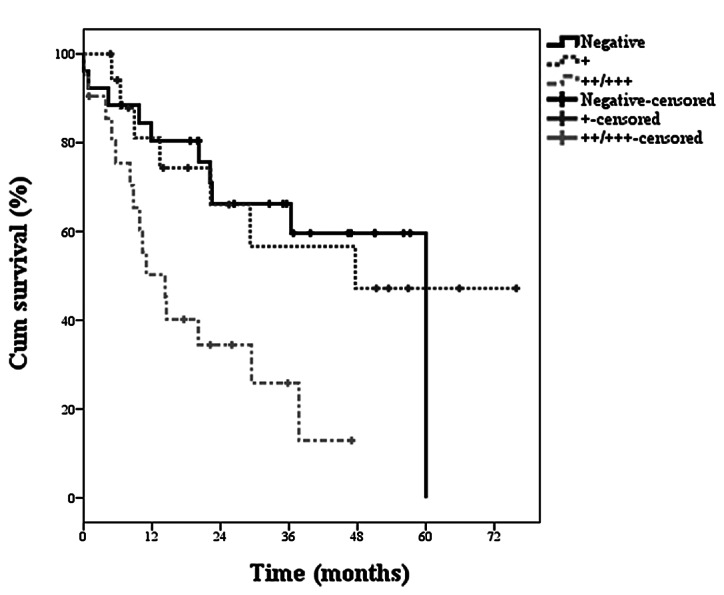
Kaplan-Meier estimate for locoregional recurrence free-survival with respect to expression of Ki-67.

Statistical analysis of local recurrence free survival (Table V) showed that the expression of Ki-67 in STS samples correlated significantly with the incidence of local recurrence in tumors. Fig. 4 presents the estimated nonparametric survival curve. Conversely, the evaluation of the expression of CD100 was not able to demonstrate significant for the event of local recurrence. Similarly, there was no significance to the event of local recurrence for the variables: chemotherapy, radiotherapy, surgery type, gender, ethnicity and pretreatment. In addition, the locoregional recurrence-free survival (Table V), the expression of Ki-67 was again significant for the incidence of local recurrence and/or regional STS and estimation of the survival curve is shown in Fig. 5. Similarly, the evaluation of the expression of CD100 was not able to show significance for the event of recurrence. The other variables (chemotherapy, radiotherapy, surgery type, gender, ethnicity and previous treatment), were not able to predict the event of local recurrence and/or regional level in patients.

In order to understand the behavior set of variables was used Cox’s multiple regression model. In addition to the markers that were statistically significant considering the log-rank test were also adjusted variables: histological grade, chemotherapy, radiotherapy and pretreatment. The results of adjusting the Cox model are presented in Tables VI, VII and VIII for global survival, local disease-free survival and locoregional disease free survival, respectively. For global survival, only histological grade and CD100 were statistically significant (Table VI). Patients with histological grade I/II has 0.16 risk of death as compared to patients with histological grade III, characterizing histological grades I/II as a protective factor. In evaluating the expression of CD100, patients with low expression (+), have 0.19 risk of death as compared to patients with higher expression (++/+++). Accordingly, low expression of CD100 (+), is a protective factor when compared with increased expression, represented here as patients with two or three crosses (++/+++).

The negative expression for this marker was not significant at 0.05 and we hypothesize that this had occurred due to the low number of events associated with this result.

In the analysis of local disease-free survival and locoregional disease-free survival, only Ki-67 was a significant marker (Tables VII and VIII, respectively). In both cases, negative or low (+) Ki-67 expression is characterized as a protective factor compared with increased expression (++ or +++). For local disease-free survival, patients with negative Ki-67 expression have 0.28 times the chance of local recurrence, compared with higher expression of the molecule (++ or +++). Also, for the expression of low Ki-67 (+) the chance of local recurrence is 0.36. For locoregional disease-free survival, patients with negative Ki-67 expression have a 0.28 chance of local and/or regional recurrence as compared to those who present a higher expression of the marker (++ or +++). Low expression of the molecule (+) has a relative risk of 0.365 for locoregional recurrence.

## Discussion

A number of previous studies have aimed to establish more effective prognostic criteria for STSs using parameters associated with location and/or tumor size, histological grade or the expression of biomarkers in clinical samples (2,4). As a prognostic factor, histological grade is currently considered to be the most reliable marker for STSs (2). In the present study, histological grade showed a statistically significant (P=0.001) value for the risk of death (global disease-free survival) in patients. Histological grade is commonly analyzed to predict disease progression in STSs, was confirmed as a suitable marker for local and global survival analysis.

Previous studies have analyzed CD100 and Ki-67 levels in STSs. However, in the present study expression of CD100 and Ki-67 proteins in STSs was analyzed by immunohistochemistry in a patient population to establish their suitability as potential clinical prognostic markers. Expression of CD100 was identified to be significantly different in patients with high and decreased rates of survival (P=0.037). Ki-67 was not revealed to correlate with overall disease-free survival. This particular scenario was repeated when we evaluate the local disease-free survival that indicated significant value of histological grade and expression of CD100 for worse prognosis. Again, Ki-67 expression was not associated with local disease-free survival.

As discussed, CD100 is associated with a number of tumorigenic processes, including regulation of immune cells and angiogenesis (23). Recently, Kato *et al*(24) demonstrated that lymphocytes infiltrating pancreatic ductal adenocarcinoma overexpress Sema4D and its receptor Plexin B1. Levels of these molecules correlate with clinical factors, including poor prognosis and metastasis. In addition, Plexin B1 expression was identified to correlate with poor prognosis in ER-positive breast cancer (25). The potential of CD100 as a biomarker in STSs was confirmed in the current study (22). CD100 expression was identified to significantly correlate with global and local survival free of disease in patients, consistent with Ch’ng *et al*(22). Therefore, we conclude that CD100 expression levels are suitable for evaluation of tumors from STS patients to determine prognosis.

CD100 expression levels were not identified to predict local relapse-free survival in patients. Only Ki-67 was identified as a biomarker for prediction of disease recurrence in patients, whereby local and locoregional recurrence-free survival was revealed to correlate with Ki-67 expression in tissues. Ki-67 is expressed throughout the majority of the cell cycle and is considered to be an excellent marker of cell division (5,26). However, Ki-67 was not demonstrated to predict disease-free survival in global or local tumors. Several studies have reported Ki-67 as a prognostic factor in various tumor types (6). A number of previous studies have reported Ki-67 to be a poor prognostic factor (27,28). However, more recently the protein was identified as a marker of specific STS subtypes suitable for adjuvant therapy (29) as well as a predictive marker of distant tumor metastases (7,30). To date, a large casuistic study has not demonstrated the correlation of this cell proliferation marker with local recurrence in STSs. Only a single study with a limited number of cases of uterine sarcoma (gynecological sarcomas are not included in the current study), has identified a correlation between local recurrence and Ki-67 expression (31).

The present study demonstrates that Ki-67 is a prognostic marker of local recurrence and/or locoregional relapse in STSs and may prove a suitable tool for the clinical management of disease progression. In addition, results demonstrate that CD100 expression is an indicator of poor prognosis of STSs. The use of these markers in routine clinical pathology may be useful as an important prognostic criterion of disease progression.

## Figures and Tables

**Table I:** Distribution of histological classification.

Histological classification	n	%
Pleomorphic sarcoma	14	21.54
Leiomyosarcoma	13	20.00
Myxoid liposarcoma	12	18.46
Myxofibrosarcoma	3	4.62
Sarcoma fibromyxoid	3	4.62
No specified origin sarcoma	3	4.62
Fibrosarcoma	2	3.08
Fibrosarcoma sclerosing	2	3.08
Synovial sarcoma	2	3.08
Synovial sarcoma monophasic	2	3.08
Fibromatosis	1	1.54
Hemangioperycitoma	1	1.54
Round cell liposarcoma	1	1.54
Dediferentiated liposarcoma	1	1.54
Pleomorphic liposarcoma	1	1.54
Pleomorphic rhabdomyosarcoma	1	1.54
Clear cell sarcoma	1	1.54
Biphasic synovial sarcoma	1	1.54
Metastatic synovial sarcoma	1	1.54

**Table II:** Distribuition of Ki-67 and CD100 expression.

Variable	n	%
Ki-67		
Negative	26	40.0
+	18	27.7
++	12	18.5
+++	9	13.8
CD100		
Negative	12	18.5
+	16	24.6
++	16	24.6
+++	21	32.3
CD100 + Ki-67		
Negative/+	18	27.7
++/+++	47	72.3

CD100, cluster of differentation 100.

**Table III:** Estimation of global survival by Kaplan-Meier considering clinical variables/demographics.

			Survival probability (%)			
Variables	Cases (n)	Mortalities (n)	1 year	3 years	5 years	P-value
Chemotherapy						
No	33	13	90.9	64.5	59.6	0.436
Yes	32	15	84.4	49.2	49.2	
Radiotherapy						
No	32	13	84.4	54.7	54.7	0.861
Yes	33	15	90.9	62.6	54.8	
Type of surgery						
Amputation	20	9	75.0	52.5	52.5	0.381
Resection	44	19	93.2	62.6	56.7	
Histological grade						
I/II	27	7	100	84.1	74.2	0.001
III	38	21	78.9	40.8	40.8	
Gender						
Female	23	8	91.3	67.3	67.3	0.223
Male	42	20	85.7	54.9	46.5	
Ethnicity						
Caucasian	43	17	88.4	59.7	55.9	0.559
Other	22	11	86.4	57.8	51.4	
Previous treatment						
No	29	15	86.2	48.9	44.0	0.177
Yes	36	13	88.9	67.9	63.4	
Ki-67						
Negative	26	7	92.3	75.6	68.7	0.084
+	18	9	77.8	54.5	48.5	
++/+++	21	12	90.5	41.0	41.0	
CD100						
Negative	12	3	100	81.5	81.5	0.037
+	16	4	100	68.9	68.9	
++/+++	37	21	78.4	48.2	40.8	

CD100, cluster of differentiation 100.

**Table IV:** Estimated local disease free survival by the Kaplan-Meier considering clinical variables/demographics.

			Survival probability (%)			
Variables	Cases (n)	Recidives (n)	1 year	3 years	5 years	P-value
Ethnicity						
Caucasian	43	14	80.2	65.0	65.0	0.283
Other	22	10	75.8	59.7	-	
Gender						
Female	23	8	82.2	70.9	57.3	0.474
Male	42	16	76.6	58.3	58.2	
Histological grade						
I	27	10	85.2	70.0	65.3	0.298
III	38	14	73.8	56.0	46.7	
Type of surgery						
Amputation	20	5	80.0	72.0	72.0	0.448
Resection	44	19	78.3	59.9	52.9	
Previous treatment						
No	29	10	77.3	62.8	55.8	0.777
Yes	36	14	79.8	63.4	58.1	
Chemotherapy						
No	33	11	87.2	67.1	56.3	0.363
Yes	32	13	69.8	58.9	58.9	
Radiotherapy						
No	32	9	86.1	68.3	68.3	0.234
Yes	33	15	72.0	58.3	48.6	
Ki-67						
Negative	26	8	88.5	75.5	69.2	0.016
+	18	4	92.9	68.6	68.6	
++/+++	21	12	55.1	50.1	-	
CD100						
Negative	12	4	100	91.7	62.9	0.298
+	16	5	81.3	65.2	65.2	
++/+++	37	15	69.8	51.3	51.3	

CD100, cluster of differentiation.

**Table V:** Estimation of locoregional disease free survival by Kaplan-Meier considering clinical variables/demographics.

			Survival probability (%)			
Variables	Cases (n)	Recidives (n)	1 year	3 years	5 years	P-value
Chemotherapy						
No	33	16	77.3	52.6	33.1	0.661
Yes	32	16	64.3	49.6	00.0	
Radiotherapy						
No	32	14	78.7	51.5	20.6	0.553
Yes	33	18	63.6	49.5	40.5	
Type of surgery						
Amputation	20	7	74.7	56.9	56.9	0.541
Resection	44	25	69.2	48.8	18.8	
Histological grade						
I/II	27	15	74.1	54.5	21.2	0.649
III	38	17	68.9	48.3	40.3	
Gender						
Female	23	12	73.2	53.0	40.3	0.817
Male	42	20	69.5	49.7	00.00	
Ethnicity						
Caucasian	43	21	73.1	49.3	28.8	0.769
Other	22	11	66.0	54.2	-	
Previous treatment						
No	29	11	77.3	57.8	50.6	0.142
Yes	36	21	65.7	46.0	0.00	
Ki-67						
Negative	26	10	84.4	66.2	0.00	0.006
+	18	7	81.1	56.6	47.2	
++/+++	21	15	50.3	25.9	12.9	
CD100						
Negative	12	5	91.7	81.5	0.00	0.101
+	16	7	81.3	59.1	49.2	
++/+++	37	20	55.3	35.8	35.8	

CD100, cluster of differentiation.

**Table VI:** Estimate of relative risk by Cox multiple model for times of global survival.

				95% CI		
Variables	Cases (n)	Mortalities (n)	Relative risk	Lower	Upper	P-value
Histological grade						
I/II	27	15	0.161	0.059	0.440	<0.001
III	38	17	-	-	-	-
Chemotherapy						
No	33	13	0.552	0.241	1.260	0.158
Yes	32	15	-	-	-	-
Radiotherapy						
No	32	9	1.132	0.513	2.500	0.759
Yes	33	15	-	-	-	-
Previous treatment						
No	29	15	2.476	2.476	1.096	5.593
Yes	36	13	-	-	-	-
CD100						
Negative	12	5	0.378	0.112	1.284	0.119
+	16	7	0.192	0.063	0.586	0.004
++/+++	37	20	-	-	-	-

CD100, cluster of differentiation.

**Table VII:** Estimate of relative risk by Cox multiple model for times of local disease-free survival.

				95% CI		
Variables	Cases (n)	Recidives (n)	Relative risk	Lower	Upper	P-value
Histological grade						
I/II	27	10	0.905	0.367	2.229	0.828
III	38	14	-	-	-	-
Chemotherapy						
No	33	11	0.780	0.328	1.857	0.575
Yes	32	13	-	-	-	-
Radiotherapy						
No	32	9	0.581	0.247	1.365	0.213
Yes	33	15	-	-	-	-
Previous treatment						
No	29	10	1.029	0.439	2.410	0.947
Yes	36	14	-	-	-	-
Ki-67						
Negative	26	8	0.338	0.127	0.903	0.030
+	18	4	0.271	0.078	0.942	0.040
++/+++	21	12	-	-	-	-

**Table VIII:** Estimate of relative risk by Cox multiple model for times of locoregional disease-free survival.

				95% CI		
Variables	Cases (n)	Recidives (n)	Relative risk	Lower	Upper	P-value
Histological grade						
I/II	27	15	1.105	0.527	2.316	0.230
III	38	17	-	-	-	-
Chemotherapy						
No	33	16	0.814	0.383	1.731	0.593
Yes	32	16	-	-	-	-
Radiotherapy						
No	32	14	0.689	0.337	1.411	0.309
Yes	33	18	-	-	-	-
Previous treatment						
No	29	11	0.630	0.296	1.340	0.230
Yes	36	21	-	-	-	-
Ki-67						
Negative	26	10	0.279	0.115	0.679	0.005
+	18	7	0.365	0.140	0.951	0.039
++/+++	21	15	-	-	-	-
